# Exosomes in the gut

**DOI:** 10.3389/fimmu.2014.00104

**Published:** 2014-03-17

**Authors:** Lesley E. Smythies, John R. Smythies

**Affiliations:** ^1^Division of Gastroenterology and Hepatology, Department of Medicine, University of Alabama at Birmingham, Birmingham, AL, USA; ^2^Center for Brain and Cognition, University of California San Diego, San Diego, CA, USA

**Keywords:** exosomes, mucosa, epithelial cells, dendritic cells, neurons, immunological synapses

## Introduction to the Gut–Microbiota Paradigm

Recent studies have highlighted the importance of cross-talk between our immune systems and our gut microbiota, the complex community of over 100 trillion commensal microorganisms (bacteria, archaea, fungi, and protozoans) that resides in the human gastrointestinal tract and which numbers about 10 times the total cells in the human body (1). The gut microbiota contribute profoundly to the function and structure of the gastrointestinal mucosa, establishing a robust network that provides us with increased digestive capacity for essential nutrients and non-nutrient factors, such as vitamins. It also protects us from infection by pathogenic microbes (2). Dysbiosis, or unbalanced shifts in the composition of the microbiota, may contribute to inflammatory bowel disease and necrotizing enterocolitis in premature infants, and are also increasingly linked to rheumatoid arthritis, multiple sclerosis, diabetes, and asthma, as well as obesity (2).

The gastrointestinal tract, which is the largest mucosal surface in the body (with a surface area of about 300 m^3^ in adults), is lined by a single layer of polarized columnar epithelial cells firmly bound to one another by tight junctions and covered by a stratified mucus layer, that together provide a barrier containing the microbiota within the lumen. Cross-talk between the microbiota and immune cells of the mucosa [dendritic cells (DCs) and macrophages], communicated through this barrier, has regulated the evolution and development of our immune systems (3–6) and differentiated our ability to recognize and distinguish between beneficial and pathogenic microbes. Microbe recognition is achieved through epithelial cell and immune cell expression of germline-encoded pattern recognition receptors (PRRs) that bind discrete microbe-associated molecular patterns (MAMPS) expressed by both commensal and pathogenic microbes (7–9). PRR expression is tightly regulated on the apical and basolateral surfaces of the epithelial cells, such that binding of PRRs can activate a series of host defense reactions, including the directed release of soluble mediators, depending upon the nature of the antigen and the polarized epithelial surface communicating with the bacteria. Intestinal DCs orchestrate and direct mucosal adaptive immune responses, balancing immune tolerance to harmless antigens and effector responses against enteric pathogens (10). To facilitate these functions, populations of intestinal macrophages, and DCs, strategically located in the sub-epithelial lamina propria (11), sample luminal antigens provided by specialized epithelial cells (goblet cells) (12) or by inserting dendrites between epithelial cells into the lumen (13–15), and phagocytose pathogenic microbes that encroach into the mucosa (11). DCs expressing the mucosal marker CD103, migrate to the MLNs, where they present acquired mucosal antigenic molecules to responsive naïve T cells (16, 17), inducing the expansion of tolerogenic or effector memory T cell populations expressing the gut homing markers α4β7 and CCR9 (18, 19), that support the T cell recruitment to the lamina propria.

## The Possible Role of Epithelial Cell-Derived Exosomes in the Regulation of Adaptive Immune Responses against the Microbiota

In addition to the release of soluble mediators, epithelial cells also release a wide variety of proteins, lipids, mRNAs, and microRNAs contained within secreted nanovesicles, or exosomes, that are formed inside the secreting cells in endosomal compartments called multi-vesicular bodies (MVBs) (20, 21). Apical secretion of exosomes into the lumen may modulate the function of distant cells along the gastrointestinal tract, or regulate the homeostasis of gut microbiota, through delivery of antimicrobial products (22). Exosomes released basolaterally into the mucosa may also regulate local innate responses to invading bacteria through microbicidal activity (22). Moreover, epithelial cell-derived exosomes released into the mucosa may be taken up by mucosal DCs and transported to the MLNs, where their contents can effect the direction of mucosal adaptive immune responses, thereby directing the education of tolerogenic CD4^+^ T cell populations in conditions of homeostasis, as well as effector CD4^+^ T cells required to combat pathogenic microorganisms during microbial invasion or infection of the intestinal mucosa. Thus, intestinal epithelial cell-derived exosomes containing **α**v**β**6 integrin and food antigen are reported to induce TGF-β^+^ tolerogenic DCs and antigen-specific TGF-β^+^ T regulatory cells, whereas food antigens in the absence of exosomes, induce a Th2-skewed response (23). Conversely, exosomes contribute to protection against luminal infection with the protozoan parasite *Cryptosporidium parvum*, where activation of TLR4/IKK2 signaling and the promotion of the SNAP23-associated vesicular exocytotic process (22) induces the formation and release of exosomes into the lumen that contain epithelial cell-derived antimicrobial peptides, including cathelicidin-37 and beta-defensin 2. Inhibition of this TLR4 signaling decreases exosomal content, reducing the ability of the cell to target antimicrobial peptides against the infectious agent locally, and perhaps influencing host antigen presentation against the bacterium systemically (22) – important evidence that exosome contents are regulated by events occurring locally in the cells from which the exosomes derive. Taken together these data suggest that epithelial cell-derived exosomes play an important role, informing not only local innate immune responses, but also DC induction of adaptive immune responses, to luminal microbiota.

## The Formation and Transfer of Exosomes from the Intestinal Lamina Propria to the Mesenteric Lymph Nodes

Exosomes were first isolated from cultured cells, but are now known to be released from many cells including red blood cells, platelets, epithelial cells, lymphocytes, DCs, tumor cells (24–27). Exosome cargo is stringently regulated by the immune condition of the cells forming the vesicles, and their transfer, by direct cell–cell contact or across gap junctions or synapses, facilitates the exchange of molecular messages, even over considerable distances. In the formation of exosomes, all types of cell so far examined bud off from their plasma membranes small lipoprotein vesicles (around 80 mm) that contain a wide variety of molecules including proteins, various types of RNA including mRNAs and microRNAs, DNA, lipid, and saccharides (28). Exosomes are constructed inside MVBs within the donor cell by invagination of its membrane. A complex mechanism then uploads a number of specific molecules into the exosome as its cargo. The MVB is then trafficked to the plasma membrane with which it fuses to release the contained exosomes into “extracellular space.”

Importantly, the exosomal lipoprotein vesicular coats protect the exosome cargo from degradation (29), even from highly destructive elements such as catabolic enzymes found within phagolysosomes, thus it is likely that exosomes remain undamaged when taken up by DCs in the mucosa. Within the lymph node, transfer of cargo from the DC to the T cell is thought to involve the immune synapse (IS), which is intimately involved in antigen presentation between DCs and T cells. Exosomes are taken up within the IS by calveoli- or clathrin-dependent mechanisms (30, 31), and transported to specific loci in the receiving cell, including the perinuclear zone, where the vesicle opens and the cargo is released. A model for the transfer of exosomes across the IS may be provided by the manner by which an exosome probably crosses the synapse between neurons. The traditional picture of a synapse was that the axon terminal and the postsynaptic spine are separated by “extracellular space” filed with cytoplasm. The exosome was pictured as crossing this fluid cytoplasm. However, electron microscopy shows that such “space” is vanishingly thin. Instead, the “space” is mainly filled with astrocyte cuffs tightly packed around the central part of the synapse plus a network of nanotubes and fibers crossing the central part (32–35). This central part is specialized for the transmission of neurotransmitter molecules, and the outer ring for the transmission of exosomes (36). Astrocytes both receive (37) and bud off (38, 39) exosomes, so it is likely that exosomes cross the synapse via the astrocyte cuff and nanotubules.

Much of the load carried by exosomes consists of epigenetic material (protein transcription factors, a wide variety of RNAs, and lengths of DNA). Epigenetic material consists of four main categories: (a) molecules that act directly on DNA by promoting co-valent binding (e.g., DNA methylases and demethylases) or non-co-valent binding (e.g., protein transcription factors); (b) agents that module the accessibility of DNA by promoting co-valent binding to histones (e.g., histone methylases and acetylases); (c) mRNAs that induce *de novo* protein synthesis in the target cell; and (d) micro RNAs that bind to mRNAs and modulate their activity (28). Exosomes from different types of cell carry different patterns of these transcription factors in their loads. In many cells, such as neurons, exosome formation is closely modulated by the degree of activity of that cell. For example, activation of glutamate receptors leads to a marked increase in exosome production mediated by calcium inflow. The exact composition of exosome loads is also exquisitely sensitive to the functional condition of the donor cell. For example, when a normal cell becomes cancerous, the ingredients of the load that its exosomes carry changes dramatically (28). Furthermore, the surfaces of different types of exosomes carry different patterns of glycosylation that can act as identifying signals, so that the exosome will bind to complementary patterns of glycosylation on the correct target cell (40). Other possible identification molecules that would allow an exosome to bind to its proper target are the heparin sulfate proteoglycans (HSPGs). Exosomes have been shown to enter cells via HSPG-mediated endocytosis. Heparanse enzyme activity is required for robust enhancement of exosome secretion (41–44). Exosomes from cancer cells depend on cell-surface HSPGs for their internalization and functional activity (30).

## Conclusion

This scenario offers an exciting new paradigm. Firstly, exosomes released from the apical or basolateral surface of gastrointestinal epithelium may contribute to antimicrobial defenses in the gut lumen. Secondly, and more interestingly, exosomes may be transported to the MLN where they modulate, by the epigenetic mechanisms listed above, host adaptive responses to luminal antigens. We are thus suggesting that there are two channels of communication between intestinal epithelial cells and target T cells in lymph nodes. The first transmits information (“software”) reflecting the contents of the gut, obtained and transmitted by DCs in the manner described earlier. The second channel transmits epigenetic instructions, in particular specific miRNAs, via exosomes to the T cell, so that it can develop the optimum molecular mechanisms or reactions (“hardware”) to process the incoming “software.” A similar system is found in the nervous system (28): information about the environment is transmitted by spike codes in axons (“software”) and instructions on how to best process this software is transmitted by epigenetic molecules via contained within exosomes. Together this results in changes in the basic functions of the receiving neurons by altering the synthesis of key proteins that play an essential role in these processes.
